# A rare jejunum retroperitoneal hernia case report and literature review

**DOI:** 10.1259/bjrcr.20200037

**Published:** 2020-07-10

**Authors:** Qiling Huang, Huiqing He, Yumin Li, Junjie Zeng

**Affiliations:** 1 Department of Radiology, People's Hospital of Heyuan Guangdong Province, Heyuan, Republic of China

## Abstract

This report describes a rare case of retroperitoneal hernia and its preoperative diagnosis. Contrast-enhanced CT and multiplanar reconstruction were used to observe the hernia ring, hernia tract, contents and blood flow of a retroperitoneal hernia. The diagnosis of and conditions related to the retroperitoneal hernia were confirmed by CT soon after hospitalisation. There are no reports on the diagnosis of primary retroperitoneal hernia on CT in the literature to date, and we share this case to facilitate the accurate and timely identification of small intestine retroperitoneal hernias in the future.

## Introduction

A 65-year-old male was admitted to the hospital after experiencing “more than 4 h of whole-body pain after a car accident”. The physical examination showed that he was not intoxicated; he had a bilateral frontal contusion and a bruise approximately 4 × 4 cm in size on the right side of the waist and abdomen. His abdominal muscles were tense, with abdominal tenderness (+) and obvious hepatic, spleen and bilateral kidney pain; the examination was negative for shifting dullness, and his bowel sounds were normal. A day after admission, an enhanced CT scan was performed.

**Figure 1.**
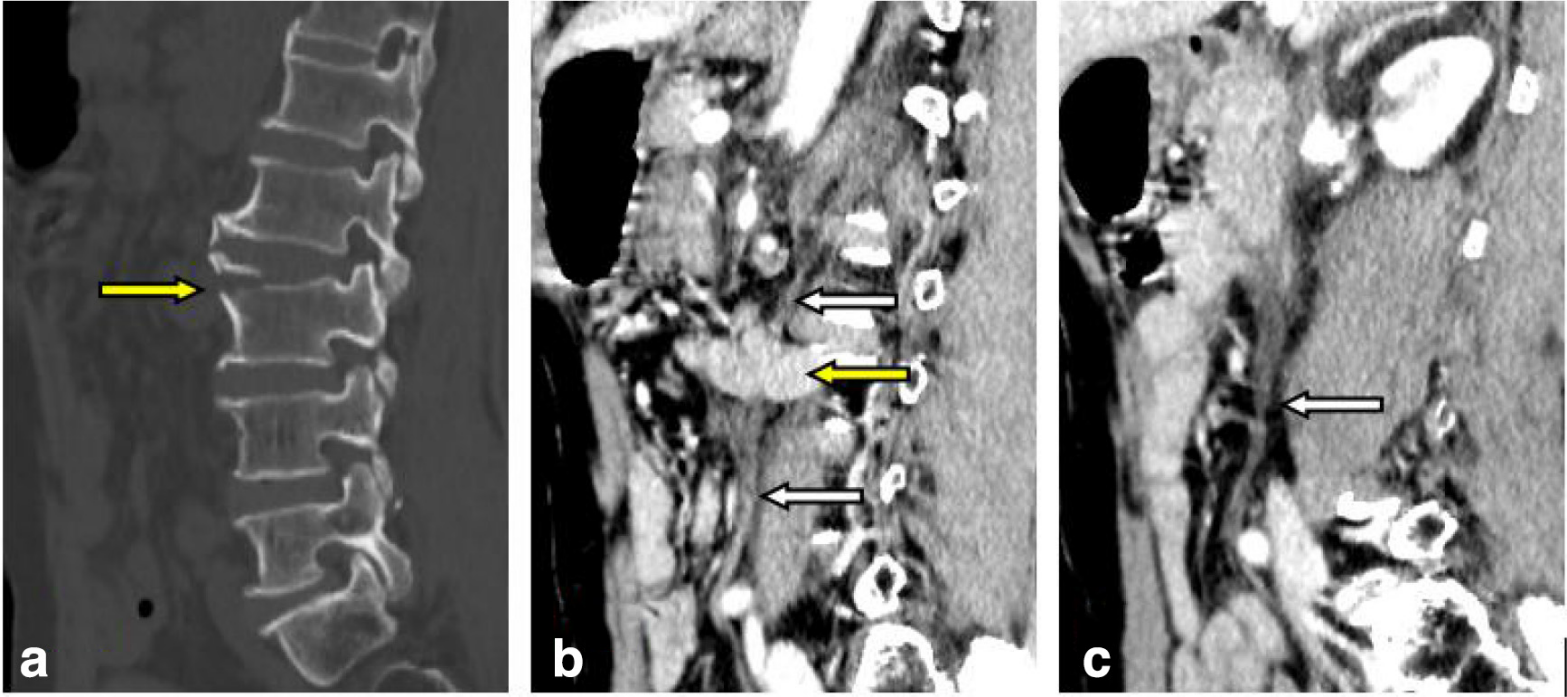
(sagittal reconstruction). a (bone window): Stretched fracture of the third lumbar vertebra, bone fragments separated slightly upward (yellow arrow). b, c: The small intestine has herniated into the retroperitoneal area behind the abdominal aorta, and the left posterior peritoneum was thickened with oedema and ruptured (white arrow).

**Figure 2.**
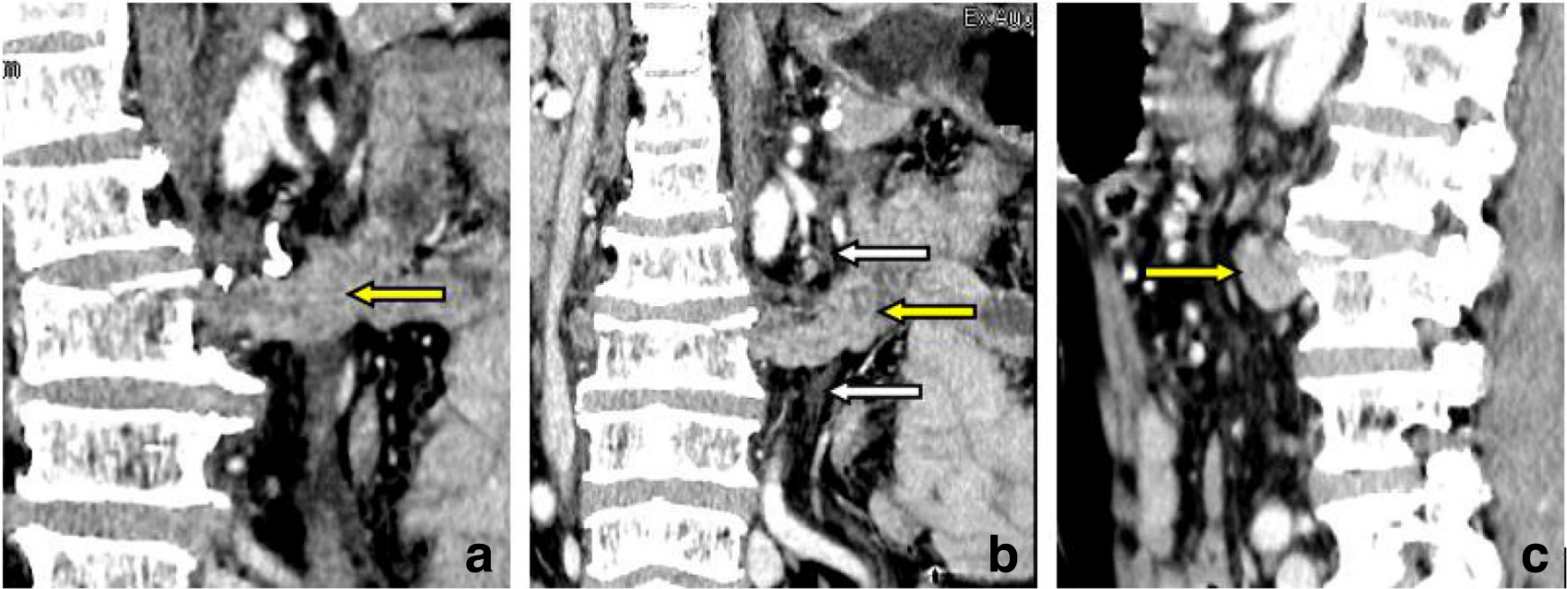
(horizontal axis). (a-c) The small intestine has protruded into the bilateral psoas-lumbar space along the left retroperitoneum (yellow arrow). (d) Re-examination the next day showed obvious oedema and thickening of the small intestine (yellow arrow).

**Figure 3.**
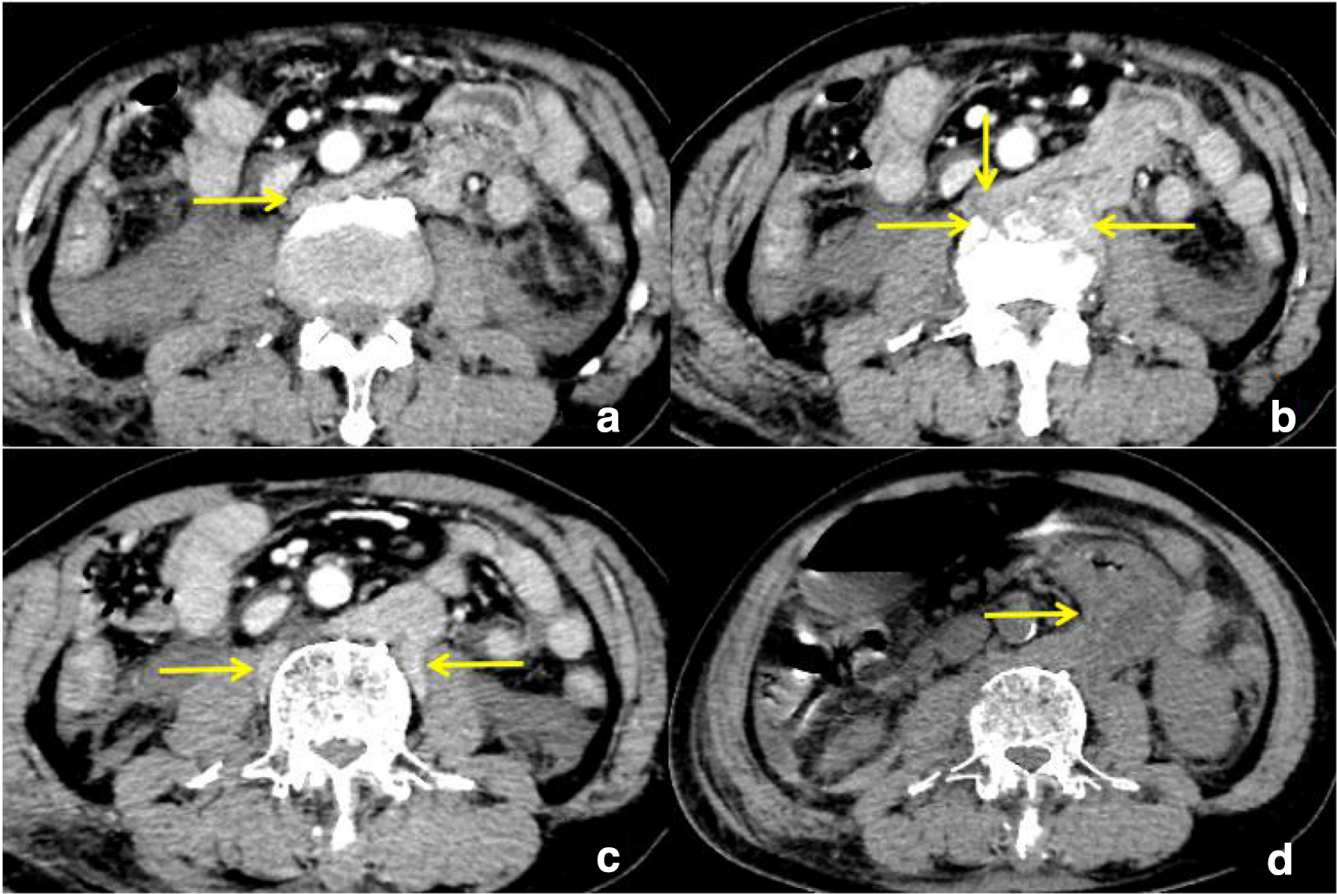
(a, b) Oblique coronal plane reconstruction; (c) sagittal reconstruction. The left posterior peritoneum was thickened with oedema and ruptured (white arrow), and the small intestine had entered the retroperitoneal space through the slit (yellow arrow).

Enhanced CT showed a stretched fracture of the third lumbar vertebra, and the fractured edge was slightly separated; an irregularity of the soft tissue was seen in the retroperitoneal area in front of the third lumbar vertebra (behind the abdominal aorta) (Figure 1), which was continuous with the duodenum and small intestine in the left upper abdomen. There was a pseudopod-like extension into the bilateral psoas-lumbar space (Figure 2a–c). Multiplanar sagittal and oblique coronal plane reconstruction showed that the left posterior peritoneum was thickened with oedema and had ruptured, and the small intestine had entered the retroperitoneal space through the slit (Figures 1 and 3). There were no obvious signs of thickening, oedema, or lumen dilatation in the small intestine wall. 1 day later, the patient's symptoms worsened, and he underwent plain CT scan. Small intestinal oedema and necrosis were found (Figure 2-d). He was immediately transferred to surgery.

## Surgical records

There was approximately 200 ml of bloody exudate in the abdominal cavity. When the exudate was cleaned, the duodenum was found to be slightly dilated from effusion, the initial jejunum was slightly dilated, and a posterior peritoneum longitudinal laceration (approximately 8 cm in length) was seen on the left anterior side of the third lumbar vertebra. The third lumbar burst fracture was approximately 20 cm away from the beginning of the Treitz ligament. The small intestine had herniated into the retroperitoneum through the laceration in the posterior peritoneum, had become embedded in the fracture stump with mesentery torsion, and could not self-reset. The bowel was reset with the assistance of an orthopaedist. Upon inspection, an approximately 6-cm-long portion of the intestines had become dark red, and congestion and oedema were obvious. The bowel canal had collapsed, peristalsis had ceased, and the corresponding mesenteric pulsation was weak. Even though the intestines were necrotic, no damage to the intestinal wall was shown. Partial bowel resection and anastomosis were performed, the spinal fractures were closed with bone wax, the retroperitoneal space was filled with a gelatine sponge, and the peritoneal rift was repaired. The post-operative diagnosis was jejunal retroperitoneal hernia with intestinal necrosis. His abdominal symptoms disappeared after 7 days, and the patient was transferred for orthopaedic treatment.

## Discussion

Intra-abdominal hernia involves protrusion of the organs or mesentery through normal or abnormal pores or fissures, leaving their original position and entering an abnormal anatomical space.^1^ Intra-abdominal hernias are divided into primary and secondary types. Primary intra-abdominal hernia is a large and deep peritoneal crypt caused by congenital factors such as abnormal intestinal rotation or peritoneal attachment during embryonic development, with defects in the omentum or mesentery, or an excessively large foramen of Winslow; subsequently, organs herniate and enter these congenital intra-abdominal spaces. Secondary intra-abdominal hernias usually originate from pathological openings formed after abdominal surgery, trauma and infection; one example is intestinal hernias, which are mainly found after endoscopic or open abdominal surgery. The formation of an intestinal hernia after a colonic mesentery defect is common and is caused by major gastric resection, radical surgery for rectal cancer, and nephrectomy.^2–8^ Intra-abdominal hernias usually lead to intestinal obstruction and intestinal strangulation after delayed diagnosis due to atypical clinical symptoms; the failure to resolve the obstructed circulation can be life-threatening because of intestinal necrosis, and the mortality rate can reach more than 50%.^9^ Therefore, early diagnosis and prompt surgery are vital. In this case, the causative factors and hernia site were extremely rare, and timely diagnosis before surgery was very difficult. To our knowledge, there has been no report in the literature of a clear diagnosis of retroperitoneal hernia before surgery.

The posterior peritoneum is relatively rigid and fixed, with more fascial layers, and the small intestine is an internal organ of the peritoneum. Therefore, retroperitoneal intestinal hernia is very rare. It is mainly secondary to peritoneal injury after laparoscopy or open nephrectomy, and most cases occur on the left side.^10–13^ Those that have occurred on the right side were seen in the foramen of Luschka.^14^ Kar et al^15^ reported a case of a primary hernia from the ileum through the parasigmoid sulcus into the retroperitoneal space. A retroperitoneal hernia secondary to a lumbar fracture is rare. Lu et al^16^ reported a case of small bowel retroperitoneal hernia after a lumbar spine fracture; a diagnosis was established when necrosis occurred in the herniated small intestine. Perforation with retroperitoneal abscess after 14 days can be treated only with extensive surgery and intensive care. Herniation into the psoas muscle-lumbar vertebra space and through the paravertebral space of the fractured bone gap, as observed in our patient, are extremely rare, and, to our knowledge, there have been no relevant published reports of this finding. This presentation is usually misdiagnosed because the incidence of retroperitoneal hernia is extremely low, the clinical manifestations lack specificity, and it is easily masked by other injuries. The peritoneal cavity and posterior peritoneal structure are complicated, and it is difficult on CT to identify the abdominal ligaments, mesentery, and peritoneum. The small intestine has relative freedom of movement, and its position is variable, making the pre-operative diagnosis even more difficult. To our knowledge, there are no reports of the diagnosis of retroperitoneal hernia on CT to date. Retrospective analysis of the clinical and imaging characteristics of this case can help clinicians improve their understanding and achieve an early diagnosis. (1) Injury mechanism: Violent stretching tends to cause excessive tension in the third lumbar ligament, leading to avulsion of the upper edge of the vertebra and tearing of the paravertebral ligament and fascia. (2) The patient had a thin body shape, and the retroperitoneal fat and lower part of the perirenal space were thin and weak, which made spinal overextension and tearing under violent stretching more likely. (3) The intra-abdominal pressure in the left upper abdomen increased sharply when the direct violent impact caused rapid deceleration, and the jejunum directly entered the retroperitoneum and herniated into the psoas muscle-lumbar intervertebral space along the torn paravertebral ligament. (4) The small intestinal mesentery was attached to the left side of the second lumbar vertebra, and the jejunum mesentery was the shortest; shear force caused the mesentery to suddenly pull the jejunum toward the attachment site. This is another anatomical factor suggesting why jejunum retroperitoneal hernia is more likely to occur on the left side than on the right side. (5) An enhanced CT scan showed that the jejunum was directly herniated into the posterior peritoneum; the multiplanar sagittal plane reconstruction showed posterior peritoneal thickening, oedema and rupture, and the enhanced CT scan enabled the posterior peritoneum, hernia contents, hernia fissure, hernia canal and hernia sac to be accurately distinguished (Figures 1, 2, 3a–c). (6) Indirect signs included mesenteric vessels that showed a spiral, twisted shape; oedema and necrosis of the small intestine inside and outside the hernia sac; proximal small bowel obstruction; and fascia exudation inside and surrounding the hernia sac. (7) Blood circulation disorders can occur in the small intestine inside the hernia sac or in the proximal and distal small intestines outside the hernia sac. Because the mesenteric vessels are herniated together, the small intestine blood circulation inside or outside the hernia sac can be affected simultaneously or separately.

In summary, retroperitoneal hernias often occur in patients with posterior peritoneal tears caused by left nephrectomy and trauma, and patients with posterior peritoneal tears caused by lumbar fractures are extremely difficult to diagnose early. Multislice spiral CT has extremely high resolution and a variety of available post-processing technologies, and it is best able to display direct and indirect signs. Enhanced CT scanning can improve tissue contrast and resolution and can provide great assistance with regard to the accurate diagnosis of intra abdominal hernia. To avoid delays in treatment, patients with lumbar fractures and abdominal pain should undergo multiplane reconstruction for careful investigation of the posterior peritoneum; when small bowel obstruction occurs, a detailed understanding of obstruction can enable the prompt detection of a retroperitoneal hernia, reduce the risk of surgical injury and death, and improve the prognosis.

## Learning point

1. Stretched lumbar fractures may lead to retroperitoneal hernia of the small intestine, especially for thin patients.
2. CT multiplanar reconstruction can confirm the diagnosis of retroperitoneal hernia, including hernia channel, hernia sac, contents, and blood flow disorder, and facilitate timely clinical management.
